# Mid-life occupational grade and quality of life following retirement: a 16-year follow-up of the French GAZEL study

**DOI:** 10.1080/13607863.2014.955458

**Published:** 2014-09-15

**Authors:** Loretta G. Platts, Elizabeth Webb, Marie Zins, Marcel Goldberg, Gopalakrishnan Netuveli

**Affiliations:** ^a^ Institute of Gerontology, Department of Social Science, Health & Medicine, King's College London, London, UK; ^b^ Department of Primary Care and Public Health, International Centre for Life Course Studies in Society and Health, Imperial College London, London, UK; ^c^ Department of Epidemiology and Public Health, International Centre for Life Course Studies in Society and Health, University College London, London, UK; ^d^ Population-based Epidemiologic Cohorts Unit, Inserm UMS 011, Villejuif, France; ^e^ Versailles St-Quentin University, UMS 011, Villejuif, France; ^f^ Institute for Health and Human Development, University of East London, London, UK

**Keywords:** life course, CASP-19, social position, Third Age

## Abstract

**Objectives:** This article aims to contribute to the literature on life course influences upon quality of life by examining pathways linking social position in middle age to quality of life following retirement in French men and women.

**Method:** Data are from the GAZEL cohort study of employees at the French national gas and electricity company. A finely grained measure of occupational grade in 1989 was obtained from company records. Annual self-completion questionnaires provided information on quality of life in 2005, measured with the CASP-19 scale, and on participants’ recent circumstances 2002–2005: mental health, physical functioning, wealth, social status, neighbourhood characteristics, social support and social participation. Path analysis using full information maximum likelihood estimation was performed on 11,293 retired participants.

**Results:** Higher occupational grade in 1989 was associated, in a graded relationship, with better quality of life 16 years later. This association was accounted for by individuals’ more recent circumstances, particularly their social status, mental health, physical functioning and wealth.

**Conclusion:** The graded relationship between occupational grade in mid-life and quality of life after labour market exit was largely accounted for by more recent socio-economic circumstances and state of health. The results support a pathway model for the development of social disparities in quality of life, in which earlier social position shapes individual circumstances in later life.

## Introduction

In advanced industrial economies, older people's lives have been transformed by growing prosperity, increased consumption and lengthening longevity in good health, while effective retirement ages have been decreasing (Gilleard & Higgs, 2002). Opportunities have expanded for people in later life to experience a new, personally rewarding period of leisure, the Third Age, marked by a lengthy period of freedom from external demands on their labour power (Laslett, 1991). However, this vision of ageing is limited to individuals able to retire from the labour market, generally men, who hold sufficient economic resources and are in good health (Bury, 1995). In other words, whether a retired person can experience the Third Age is related to their current location within the social structure, a location which has itself been shaped by the individual's personal history (Blane, Higgs, Hyde, & Wiggins, 2004; Hertzman & Power, 2006). Taking such a life course perspective, which examines the interplay between the social structure, individual development and time, highlights how earlier exposures can exert long-term effects upon health and well-being (Elder, Johnson, & Crosnoe, 2003). More concretely, exposure to economic and social advantage in mid-life may place an individual onto pathways leading to a better state of finances and health following retirement, which in turn improve their chances of experiencing a flourishing and independent period of later life.

Research employing life course perspectives to examine how individual histories relate to quality of life or well-being in later life is at an early stage of development (George, 2010; Layard, Clark, Cornaglia, & Powdthavee, 2013). While long-term effects of earlier circumstances upon quality of life and well-being have been observed, particularly in relation to social position in childhood or mid-life (cf. Niedzwiedz, Katikireddi, Pell, & Mitchell, 2012, for a recent review), these effects tend to be small compared to the size of life course influences upon health (Blane et al., 2004) or in relation to the influence of current factors (Blane, Webb, Wahrendorf, & Netuveli, 2012). In contrast, contemporaneous influences upon older people's quality of life have been extensively documented. A consensus is emerging that the main predictors of quality of life in later life are: physical and mental health, participation in social activities, social relationships, neighbourhood quality and financial adequacy (Layte, Sexton, & Savva, 2013; McMunn, Nazroo, Wahrendorf, Breeze, & Zaninotto, 2009; Netuveli, Wiggins, Hildon, Montgomery, & Blane, 2006; Siegrist & Wahrendorf, 2009; Von dem Knesebeck, Wahrendorf, Hyde, & Siegrist, 2007; Webb, Blane, McMunn, & Netuveli, 2011; Wiggins, Higgs, Hyde, & Blane, 2004; Zaninotto, Breeze, McMunn, & Nazroo, 2013; Zaninotto, Falaschetti, & Sacker, 2009). Each of these factors might represent a pathway indirectly linking earlier circumstances to subsequent quality of life. Such pathways or chains of risk are an area of interest in life course theory (Kuh, Ben-Shlomo, Lynch, Hallqvist, & Power, 2003), with studies suggesting that previous conditions might affect quality of life via their influence upon individuals’ current circumstances, particularly health, social support and socio-economic position (Blane et al., 2004; Read & Grundy, 2011).

An individual's social position conditions their life chances; it could therefore be expected that mid-life social position would affect individuals’ quality of life following retirement, perhaps by affecting area of residence, state of health, social support or pension provision in later life (Blane, Netuveli, & Bartley, 2007). To our knowledge, only two studies, both using British data, have examined the relationships between mid-life social position and quality of life after retirement in terms of possible pathways: one study suggested intermediary roles for physical functioning and subjective financial situation (Blane et al., 2012) and the other proposed a role for subjective social status (Netuveli & Bartley, 2012).

Therefore, this study will extend previous life course research by examining in the French GAZEL cohort, a large, prospective sample, whether social position in mid-life is associated with quality of life following retirement, using a linear measure of mid-life social position: occupational grade. This lengthy series of grades is a multi-dimensional measure of social position, which, like other occupational classifications, will capture aspects of social status, wages and autonomy (CFDT, 2013; Marmot & Shipley, 1996).

In a second stage, a pathway model of the development of disparities in quality of life will be constructed. With it, we will examine whether recent circumstances account for any association between mid-life social position and quality of life following retirement. A range of proximal determinants of individuals’ quality of life have been selected to represent possible pathways via more recent circumstances, including health, social status, economic circumstances, neighbourhood characteristics and social relationships. A structural equation modelling (SEM) approach will be taken in order to model all of the pathways simultaneously, as well as adjusting for interrelationships between the measures of recent circumstances.

The study aims to answer three research questions:

1. Is there a gradient between mid-life social position and quality of life following retirement?
2. To what degree is the association of earlier social position with quality of life accounted for by pathways via more recent circumstances?
3. Which specific pathways are implicated?

We predict that higher previous social position will be associated with better quality of life following labour market exit, and that more recent circumstances will account for these differences, particularly health and financial characteristics.

## Methods

### Data

The analyses were performed using the GAZEL occupational cohort of persons employed by the French national gas and electricity company (EDF-GDF). At study onset, in 1989, 15,011 male employees aged 40–50 years and 5614 female employees aged 35–50 years were recruited (Goldberg et al., 2007). The data-set combines detailed administrative data on occupational histories with information obtained from annual self-completion questionnaires completed by participants since 1989. Although the GAZEL cohort represents a specific employment sector, the study population was recruited from urban and rural areas throughout France, represents a wide range of occupations and has a socio-economic structure that compares well to the French population (for a detailed cohort profile, see Goldberg et al., 2007). The GAZEL participants benefited from civil-servant type posts with secure employment conditions, and consequently largely had uninterrupted careers and relatively comfortable pensions, including those who worked in low-skilled occupations (Chevalier, Leclerc, Blanc, & Goldberg, 1987). Attrition rates are low, with about 0.5% of the employees lost to follow-up, and about three-quarters of the cohort responding to each annual questionnaire (Zins, Leclerc, & Goldberg, 2009).

Certain cohort members were excluded from this analysis: by 1 January 2005, 909 participants had died and 3093 individuals had not yet retired. An additional six participants were excluded because they were already retired in 1989 when occupational grade was recorded. Additionally, participants were excluded if information on their occupational grade in 1989 was missing (26 participants) or they did not provide CASP-19 quality of life scores in 2005 (5298 participants). Following these exclusions, the sample comprised 11,293 men and women.

## Measures

### Quality of life

Subjective quality of life was evaluated using the CASP-19 scale from the 2005 self-completion questionnaire. CASP-19 is a validated measure of quality of life specifically designed for individuals in later life and which has been used in a wide range of ageing surveys (Hyde, Wiggins, Higgs, & Blane, 2003; Netuveli et al., 2006). It avoids reducing older people's quality of life to their experience of health (Hyde et al., 2003). Instead, drawing on the theoretical perspective of the satisfaction of human needs (Doyal & Gough, 1991), and the sociological literature as it pertains to ageing and the self as a reflexive project (Giddens, 1991; Gilleard, 1996), the scale measures the fulfilment of those human needs which are particularly relevant in later life. These needs, Control, Autonomy, Self-realization and Pleasure, form the acronym CASP. The first two domains emphasize older people's abilities to be free from undue interference and to intervene in their environment (Wiggins et al., 2004); the latter two domains, closely related to the Third Age (Laslett, 1991), measure the degree to which individuals pursue reflexive projects of self-realization through activities that make them happy (Higgs, Hyde, Wiggins, & Blane, 2003). Each of the 19 four-point Likert-scaled items in the CASP-19 measure draws on one of the four domains, which are summed into a single total score assessing quality of life. The full range of the scale is 0–57 and higher scores indicate better quality of life. In the study sample, the skewness of the CASP-19 scale was –0.96 and its kurtosis was 4.09. Technical details about the CASP-19 scale and results of validation procedures are provided elsewhere (Hyde et al., 2003; Wiggins, Netuveli, Hyde, Higgs, & Blane, 2008).

### Demographics

Company personnel files indicated participants’ gender, date of birth and retirement date. Date of birth was used to calculate each participant's age in 1989. Age was centred at 45 years and a squared age term was generated in order to model non-linear relationships with age. Each participant's retirement date was used to calculate the number of completed years they had been retired by the beginning of 2005. The scale runs from 0 to 15, where 0 indicates that the participant retired during 2004, 1 that they retired during 2003 etc. Finally, a variable indicating each participant's age in years at retirement was generated from dates of birth and retirement recorded in the EDF-GDF personnel files.

### Occupational grade

Occupational grade was used to measure social position; such gradings have been found to precisely define occupational hierarchies and are excellent indicators of mid-life social position (Hoven & Siegrist, 2013; Marmot et al., 1998). Participants’ grades in 1989 were extracted from the EDF-GDF personnel records.

At EDF-GDF, occupational grade is an internal company classification in which all jobs are classified into 23 grades arranged on a 1–60 scale, where higher values represent a higher hierarchical position, associated with a larger salary, greater autonomy and responsibility in work tasks and higher status within the company (CFDT, 2013). Each occupational grade contains people holding a wide range of job titles and most posts are distributed across a range of grades. Both blue and white-collar occupations are found at each grade, apart from the uppermost grades, containing the most senior managers, who have both blue and white-collar career histories.

In our sample in 1989, only the grades 1–52 were represented. The median grade overall was 8, a level represented by a wide range of occupations including: higher level secretaries, printing assistants, draftsmen, maintenance technicians in electricity, nurses, welders and warehouse supervisors. For the SEM, the variable was converted from 1–52 to 1–21 to prevent the highest grades from becoming outliers (skewness = 0.55, kurtosis = 3.48).

### Predictors of quality of life

Seven factors that are known to predict quality of life and for which there is evidence of associations with social position were included in the analyses as mediating variables: physical health, mental health, wealth, perceived social status, neighbourhood characteristics, social support and social participation.

#### Mental health

The mental health sub-scale from the French standard version of the internationally validated Short-Form 36 Health Survey (SF-36) was used to measure mental health functioning in the 2003 questionnaire (Leplège, Ecosse, Verdier, & Perneger, 1998; Ware Jr & Sherbourne, 1992). The mental health scores ranged from 0–100, with higher scores indicating better health (skewness = –0.81, kurtosis = 3.54).

#### Physical functioning

The physical functioning sub-scale from the French version of the SF-36 survey was taken from the 2003 questionnaire. The range of the physical functioning scores was 0–100, with higher scores indicating better functioning (skewness = –2.67, kurtosis = 12.95).

#### Wealth

In the 2002 questionnaire, participants were asked about the net worth of the household, specifically: ‘If you sold all of your assets (main residence, secondary residence, furniture, car, jewellery and after repaying any debts), what value would this correspond to?’ Participants selected one of nine response categories, ranging from less than 1525 euro (10,000 French francs) to 457,347 euro (3 million French francs) or more. In this age group, where incomes can be low or absent, wealth is considered a better measure of economic status than measures based on income because it represents resources which can be drawn on retirement (Banks, Karlsen, & Oldfield, 2003; Pollack et al., 2007). Although this variable is ordinal, it has nine categories with a unimodal slightly negatively skewed distribution (skewness = –0.88, kurtosis = 5.28), and was therefore included in the structural equation model as a 0–8 continuous variable, as is recommended (Johnson & Creech, 1983; Rhemtulla, Brosseau-Liard, & Savalei, 2012).

#### Subjective social status

This variable measures individuals’ subjective sense of their standing on the social ladder in relation to multiple dimensions of socio-economic status and social position, including occupational grade, household income, education, satisfaction with standard of living and feeling of financial security (Adler, Stewart, & the Psychosocial Working Group, 2007; Singh-Manoux, Adler, & Marmot, 2003). Previous research using the Whitehall II cohort found that subjective social status was more closely related to feelings of financial security over the next 10 years than occupational grade, an objective measure of socio-economic status, suggesting that subjective social status assesses a person's resources and future prospects more closely than the objective measure (Singh-Manoux, Marmot, & Adler, 2005).

Specifically, in the 2004 questionnaire, cohort members were asked to mark an X to show where they stood on a drawing of a ladder, anchored on the uppermost rung J by those who are most advantaged in terms of income, education and occupation and at the bottom on rung A by the worst off. Individuals who ticked more than one rung had their responses averaged. This version of the MacArthur Scale of subjective social status yielded a 0–9 score (skewness = –0.10, kurtosis = 3.03) which was used as a continuous variable.

#### Neighbourhood characteristics

Two questions in the 2003 questionnaire measured cohort members’ satisfaction with access to (1) local shops and services and (2) leisure activities. Dissatisfaction with access to either set of amenities was coded as 0, otherwise 1. Some cohort members had moved away from their neighbourhoods by 2005, when quality of life was measured. Consequently, participants’ postcodes were examined and if these had changed 2003–2005, which concerned 844 participants in the study sample, the values were converted to missing.

#### Social support

Social support was measured in the 2004 questionnaire by the individual's subjective evaluation of whether they had a close, confiding relationship. The question used was: ‘Is there somebody with whom you can discuss personal things or who can help you take an important decision?’ Negative responses were coded as 0, affirmative responses were coded as 1.

#### Social participation

In the 2005 questionnaire, participants were asked how often they participated in a range of social activities, from a list of: (1) sports and other clubs, (2) union and political activities, (3) volunteering or charitable activities, (4) communal religious activities and (5) following a course or a training programme. We coded the response categories as follows: ‘nearly every day’ (3 units), ‘nearly every week’ (2 units), ‘less frequently’ (1 unit) or ‘never’ (0 units). These units were summed across all the activities to create a single score indicating each individual's overall level of social participation. A three-level categorical variable was subsequently created, which was converted into dummy variables. The reference group contained participants gaining the minimum score of 0 units because they reported no participation in any social activities. One dummy contained participants with scores of 1–3 units indicating low–medium levels of participation; the other dummy contained participants scoring ≥4 units indicating relatively high social participation.

### Auxiliary variables

Auxiliary variables were included in the analyses in order to improve the performance of the full information maximum likelihood (FIML) estimation procedure (Graham, Cumsille, & Elek-Fisk, 2003). A set of questionnaire items from the baseline survey in 1989, which have been shown to predict attrition and therefore might be influencing the process causing the missing data (Goldberg, Chastang, Zins, Niedhammer, & Leclerc, 2006; Sterne et al., 2009), were included as auxiliary variables: education level, self-rated health, smoking status and alcohol use. In addition, if repeated measures from other waves were available, they were also included as auxiliary variables: CASP-19 from the 2009 questionnaire as well as mental health and physical functioning from 2007. Including such variables, which are highly correlated with the missing variables, can reduce the bias that might otherwise be generated by the missing data (CASP-19 in 2009 and 2005 were correlated at 0.74; mental health in 2003 and 2007 at 0.61; physical functioning in 2003 and 2007 at 0.60) (Collins, Schafer, & Kam, 2001).

### Statistical analysis

In a first step, the data were analysed descriptively, using Stata 13.1 (StataCorp, 2012), to display the characteristics of the 11,293 sample members. In order to answer the first research question, examining the relationship between mid-life social position and quality of life following retirement, mean quality of life scores in 2005 were contrasted by occupational grade in 1989. A test for trend was performed, using the non-parametric test for trend across ordered groups.

To answer the second research question, SEM was performed using the Mplus 7 program (Muthén & Muthén, 2012). Paths between observed variables were calculated and direct and indirect effects from mid-life social position upon quality of life were estimated, assuming a multivariate normal distribution for the endogenous variables (Kline, 2005, pp. 112–114). In order to deal with sample attrition, the models were estimated using FIML estimation with auxiliary variables that predicted missingness or were predictive of the missing values themselves in a saturated correlates model (Graham et al., 2003; Spratt et al., 2010).

**Figure 1.**
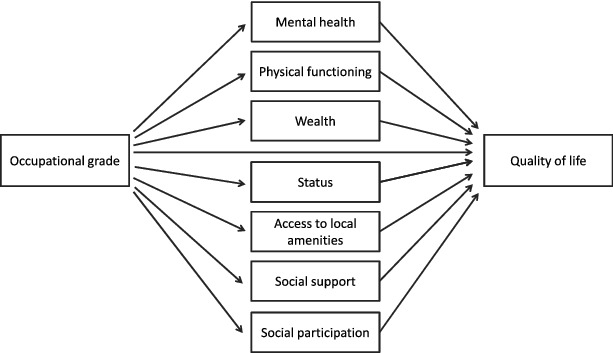
Postulated paths connecting occupational grade in 1989 with quality of life 16 years later.

A just identified model was created in which occupational grade could have an effect on quality of life 16 years later either directly or indirectly via one or more of the pathways of: mental health, physical functioning, wealth, perceived social status, accessibility of local amenities, social support and social participation (Figure 1). Gender, age, age-squared, age at retirement and years since retirement were included in the model as control variables. The analyses controlled for years since retirement because it is possible that the relationship between circumstances at work and subsequent quality of life is affected by time since labour market exit (Hyde & Jones, 2007).

## Results

### Descriptive findings

Table 1 displays univariate statistics and describes the amount of missing data from the study sample. Highest proportions of values were missing from variables obtained from the self-completion questionnaires, at around 17% for the wealth variable which had the most values missing. The GAZEL cohort members were mostly male (80.7%) and were aged 36–50 years in 1989. This corresponds to 52–66 years in 2005, when participants had been retired for 5.3 years on average.

**Table 1.** Descriptive statistics for the study sample of 11,293 GAZEL cohort participants.

Variable (year)	Category or range	Percent or mean (std dev.)	Number in category	Number missing
CASP-19 quality of life (2005)	6–57	43.5 (7.6)	–	0
Gender				0
	Male	80.7%	9114	–
	Female	19.3%	2179	–
Age (1989)	36–50	45.0 (3.0)	–	0
Years since retirement (2005)	0–15	5.3 (3.3)	–	0
Age at retirement	35–62	54.4 (2.6)	–	0
Mental health (2003)	0–100	70.2 (16.8)	–	686
Physical functioning (2003)	0–100	90.2 (12.8)	–	634
Wealth (2002)				1914
	<1525 euro	0.6%	53	–
	1525 to <4574 euro	0.3%	32	–
	4574 to <7623 euro	1.6%	150	–
	7623 to <15,245 euro	2.0%	188	–
	15,245 to <76,225 euro	10.1%	949	–
	76,225 to <152,449 euro	23.5%	2206	–
	152,449 to <304,898 euro	40.6%	3807	–
	304,898 to <457,347 euro	14.8%	1383	–
	≥457,347 euro	6.5%	611	–
Subjective social status (2004)	0–9	4.8 (1.4)	–	769
Satisfaction with access to local amenities (2003)				1433
	Unsatisfied	21.8%	2146	–
	Satisfied	78.2%	7714	–
Social support (2004)				575
	No	14.2%	1521	–
	Yes	85.8%	9197	–
Social participation (2005)				1213
	None	21.9%	2203	–
	Low–medium	39.5%	3981	–
	High	38.7%	3896	–
Occupational grade (1989)	1–21	9.0 (3.3)	–	0
				
Auxiliary variables				
CASP-19 quality of life (2009)	9–57	43.2 (7.5)	–	1704
Mental health (2007)	0–100	70.0 (16.7)	–	952
Physical functioning (2007)	0–100	88.8 (14.2)	–	823
Self-rated health (1989)	0–7	1.91 (1.27)	–	85
Smoking status (1989)				65
	Current smoker	25.3%	2843	–
	Non-smoker or ex-smoker	74.7%	8385	–
Alcohol consumption (1989)				68
	Abstainers and heavy drinking	10.7%	1206	–
	Occasional drinking	56.5%	6347	–
	Moderate drinking	15.9%	1784	–
	Average drinking	16.8%	1888	–
Education level (1989)				188
	University	15.8%	1754	–
	High school or other	8.9%	987	–
	Less than high school	75.3%	8364	–

The statistics presented in this table for variables where values are missing correspond to the values participants provided (in contrast, mean values generated through FIML estimation are displayed in Table A1). Individuals tended to place themselves approximately mid-way on the subjective social status scale (mean = 4.8, standard deviation = 1.4) and reported mean quality of life scores of 43.5 units (standard deviation = 7.6). In terms of the mediating variables, most respondents reported having a close, confiding relationship (85.8%), being satisfied with access to local amenities (78.2%) and being involved in organized social activities to some degree (7877 participants, or 78.1%). Physical functioning scores were higher on average than mental health scores.

### Bivariate associations

Table 2 displays correlations between all of the variables. Higher quality of life scores were reported by individuals in better physical and mental health, who had greater wealth and social status, a higher age at retirement and who reported satisfaction with access to local amenities, a confiding relationship and high social participation. Greater age, more years since retirement, female gender and low–mid levels of social participation (compared to no social participation) were associated with poorer quality of life.

**Table 2.** Correlation matrix for the variables used in the analysis for the GAZEL cohort study sample (*n* = 11,293).

	1	2	3	4	5	6	7	8	9	10	11	12	13	14	15
1. CASP-19 (2005)	1.000														
2. Gender	−0.089*	1.000													
3. Age-centred	−0.039*	−0.143*	1.000												
4. Age-squared	−0.036*	0.226*	−0.035*	1.000											
5. Years since retiring (2005)	−0.060*	−0.048*	0.663*	0.184*	1.000										
6. Age at retirement	0.027*	−0.116*	0.335*	−0.276*	−0.471*	1.000									
7. Mental health (2003)	0.538*	−0.203*	0.032*	−0.050*	0.008	0.028*	1.000								
8. Physical functioning (2003)	0.336*	−0.179*	−0.074*	−0.037*	−0.077*	0.009	0.298*	1.000							
9. Wealth (2002)	0.158*	−0.054*	0.049*	−0.010	−0.027*	0.089*	0.098*	0.092*	1.000						
10. Social status (2004)	0.252*	−0.033*	0.054*	0.001	−0.083*	0.160*	0.141*	0.122*	0.338*	1.000					
11. Local amenities (2003)	0.138*	−0.021*	0.014	0.018*	0.003	0.014	0.120*	0.080*	0.045*	0.059*	1.000				
12. Social support (2004)	0.190*	0.022*	−0.022*	0.016	−0.006	−0.020*	0.155*	0.047*	0.039*	0.075*	0.052*	1.000			
13. Low–mid participation (2005)	−0.044*	0.005	0.004	0.012	0.016	−0.016	−0.006	−0.013	0.004	−0.043*	−0.018*	−0.008	1.000		
14. High participation (2005)	0.139*	−0.041*	0.007	−0.029*	−0.020*	0.028*	0.063*	0.083*	0.034*	0.098*	0.015	0.061*	−0.641*	1.000	
15. Occupational grade (1989)	0.131*	−0.248*	0.178*	−0.058*	−0.035*	0.246*	0.111*	0.112*	0.293*	0.482*	0.003	0.006	−0.036*	0.090*	1.000

Note: *p < 0.05.

Occupational grade in 1989 was positively correlated with quality of life in 2005. Female gender and more years since retirement were associated with lower occupational grade; greater age and higher age at retirement were positively correlated with occupational grade. Occupational grade correlated with all of the potential mediating variables, except for satisfaction with access to local amenities and social support.

**Figure 2.**
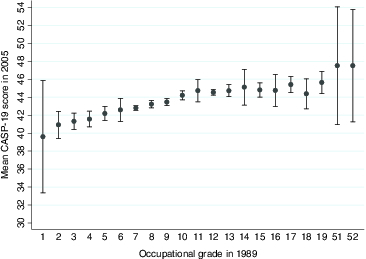
CASP-19 quality of life scores in 2005 by occupational grade in 1989 for 11,293 participants in the GAZEL cohort.

In order to answer the first research question, concerning the existence of a graded association between occupational grade and quality of life, Figure 2 displays mean quality of life in 2005 in relation to occupational grade in 1989. It shows a graded relationship between occupational grade in 1989 and quality of life 16 years later following retirement, with a difference of more than five units between participants who were employed in the lowest (1–3) and highest grades. The test for trend was significant at *p* < 0.001, indicating that there appears to be a trend across the ordered groups.

If the relationship is modelled as linear, a one-step increase in occupational grade is associated with an increase in the CASP-19 quality of life score of 0.30 units (95% CI = 0.26; 0.34, *p* < 0.001). An idea of the size of this effect can be obtained by comparing it with having a limitation to normal physical activities, such as difficulty dressing or needing help to walk about outside, using the physical limitations sub-scale of the Nottingham Health Profile, which was measured in 2006. Being unable to perform one normal physical activity is associated with a 3.1 unit lower CASP-19 score, which would correspond to a difference of about 10 occupational grades.

Although modelling a non-linear relationship between grade and quality of life through the inclusion of a quadratic term for grade improved the fit of the model (likelihood ratio test statistic = 9.43, *p* = 0.002), the difference was small and not significant at *p* < 0.001. Modelling a mediating non-linear relationship would greatly increase the complexity of the modelling and the difficulty of interpretation; therefore, we make the simplifying assumption of linearity and accept the consequential slight misspecification of the model.

The association is finely grained but the importance of occupational grade in predicting quality of life in retired participants 16 years later was small; only 1.7% of the variation in quality of life was accounted for by mid-life occupational grade. Both genders are presented together, but similar broad trends were observed for each gender separately (data not shown). In a sensitivity analysis regressing quality of life on occupational grade, gender, age and an interaction term between gender and occupational grade, the coefficient for the interaction was small and insignificant (–0.02, 95% CI = –0.15;0.11, *p* = 0.771).

### Path analyses

FIML estimation with auxiliary variables was used in order to include all 11,293 eligible participants in the analysis. A path model was estimated from the covariance matrix provided in Appendix Table A1 which included all direct and indirect pathways between occupational grade and quality of life and which allowed all variables to correlate.

In terms of the second research question, which concerns the existence of any direct effects of earlier social position upon quality of life, the direct path between occupational grade in 1989 and quality of life in 2005 was not significant in the model, indicating that the included indirect pathways entirely accounted for the covariation between occupational grade and subsequent quality of life (Table 3).

**Table 3.** Standardized and unstandardized coefficients for the paths between occupational grade in 1989 and quality of life in 2005 for 11,293 GAZEL cohort participants.

Path	Unstandardized estimate	Standard error	*p*-value	Standardized estimate
Indirect	0.319	0.018	<0.001	0.138
via mental health	0.069	0.010	<0.001	0.030
via physical functioning	0.034	0.004	<0.001	0.015
via wealth	0.033	0.006	<0.001	0.014
via subjective social status	0.164	0.011	<0.001	0.071
via access to local amenities	−0.001	0.001	0.481	0.000
via social support	0.004	0.002	0.095	0.002
via low–mid social participation	−0.003	0.001	0.025	−0.001
via high social participation	0.019	0.003	<0.001	0.008
Direct	−0.035	0.021	0.103	−0.015
Total	0.284	0.023	<0.001	0.123

The final research question concerned which pathways accounted for the effect of occupational grade in mid-life upon quality of life following retirement. Several variables accounted, at the 5% level, for the indirect effects of occupational grade upon subsequent quality of life: mental health, physical functioning, wealth, social status and the dummy for high social participation. Comparing the standardized estimates, about half of the indirect effect of occupational grade upon quality of life was via subjective social status (proportion of indirect effect = 51%), with most of the rest of the effect being accounted for by mental health (22%), physical functioning (11%) and wealth (10%) and a small portion by the dummy for high levels of social participation (6%).

**Figure 3.**
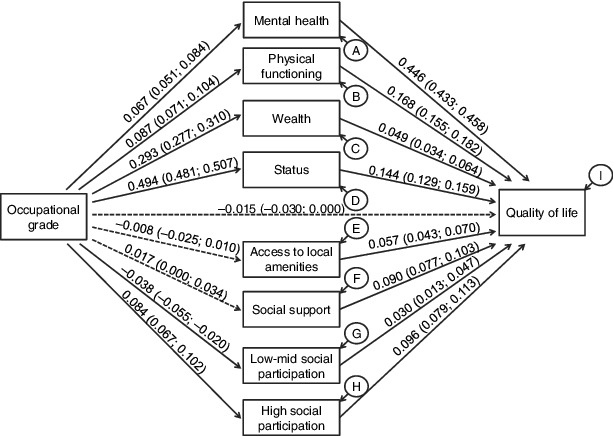
Standardized path coefficients from the FIML estimation path model for 11,293 participants in the GAZEL cohort.

Figure 3 displays standardized coefficients and 95% confidence intervals. All of the potential mediating variables were associated with quality of life in 2005. Most were also associated with earlier occupational grade at the 5% level apart from satisfaction with access to local amenities and social support. Higher occupational grade was associated with greater wealth, higher subjective social status, better physical functioning, better mental health and high social participation. The association of occupational grade with low to medium social participation was trivial because the disturbance was close to 1, suggesting that occupational grade covaried little if at all with low to medium social participation.

## Discussion

In this prospective study of retired employees from the French GAZEL occupational cohort, there was a finely graded relationship between higher occupational grade in mid-life and better quality of life 16 years later, following retirement. To our knowledge, this is a novel finding, although it corresponds to the graded relationship between a tripartite category of current or most recent occupational grade and quality of life reported in the Whitehall II cohort (Stafford, Chandola, & Marmot, 2007) and to the relationship between quality of life and previous social class reported among the retired participants from the English Longitudinal Study of Ageing (Blane et al., 2007). The relationship between occupational grade and quality of life we present here only explained 1.7% of the variability in CASP-19 quality of life, a finding which corresponds to previous reports in which the influence of life course factors upon quality of life was small (Blane et al., 2004; Wiggins, Erzberger, Hyde, Higgs, & Blane, 2007).

A range of recent circumstances, namely mental and physical health, wealth, social status, neighbourhood characteristics, emotional support and social participation, predicted quality of life in the GAZEL cohort. Similar factors have predicted quality of life (Layte et al., 2013; Netuveli et al., 2006; von dem Knesebeck et al., 2007; Wiggins et al., 2004) and change in quality of life (Webb et al., 2011; Zaninotto et al., 2009) in other, mainly British and Irish cohorts.

In results presented here, the relationship between earlier social position and quality of life following retirement was accounted for by several recent circumstances. This result provides support for a pathway model of life course influences upon quality of life; it corresponds to and extends previous work using British data-sets describing pathways to later quality of life via individuals’ material circumstances and health problems (Blane et al., 2004, 2012).

The variables which appeared to mediate the relationship between occupational grade and quality of life following retirement were: physical and mental health, wealth, social status and, more equivocally, social participation. The finding that both social status and wealth are implicated in the longitudinal relation between earlier social position and quality of life suggests that socio-economic inequalities from working life persist after retirement (Marmot & Shipley, 1996). In terms of the mediating variable of health, one manner in which occupational grade might affect subsequent quality of life is via its impact on the tasks individuals performed during their careers and therefore upon their working conditions (Hoven & Siegrist, 2013), which have been shown to impact upon their health and quality of life (Levy, Wegman, Baron, & Sokas, 2011; Ogg & Renaut, 2013; Platts et al., 2013; Siegrist, Wahrendorf, von dem Knesebeck, Jürges, & Börsch-Supan, 2007).

In contrast, social support and subjective evaluation of access to local amenities did not appear to be mediating variables. Existing evidence presents a mixed picture concerning associations between social position and social support (Gorman & Sivaganesan, 2007; Taylor & Seeman, 1999), however it might be that using a measure of social position based on social class would have predicted variations in support (Turner & Marino, 1994). The lack of association between occupational grade and satisfaction with access to local amenities is more surprising; perhaps more advantaged participants selected their neighbourhoods using other criteria. Further analyses could explore other aspects of neighbourhood characteristics.

It is necessary to embed the results obtained from the GAZEL participants in the cohort's social and geographical setting. Having a Bismarckian welfare state in which entitlements are related to previous employment, a division has emerged in France between insiders, typified by the GAZEL cohort members, who were included in the labour market and able to contribute to and benefit from social insurance systems, and outsiders receiving minimal social protection (Palier, 2010). In addition, the GAZEL participants were able to retire at an early age as a result of retirement rules providing early retirement from careers involving strenuous work; the modal retirement age being 55 years for the cohort as a whole. This means that the association between social position and wealth may be diminished in this sample, because one mechanism underpinning the association may be the increased likelihood of unemployment that people in disadvantaged social classes are more vulnerable to, especially at older ages (Bartley & Plewis, 2002). Therefore, it is possible that the relationship between social position in mid-life and subsequent quality of life might be stronger in other cohorts and the pathways slightly different.

### Strengths and limitations

This study benefits from a number of strengths. It is a prospective study using a large cohort that provides a representative range of occupations found in French society, including blue-collar jobs (Goldberg et al., 2007). One of few studies examining quality of life from a life course perspective, it extends this approach to a continental European country. Quality of life is measured with the multi-dimensional CASP-19 measure, a theoretically based measure with good psychometric properties (Bowling & Stenner, 2011) and social position is measured with a fine-grained occupational scale obtained from company records.

However, this study has a number of limitations. First, although the model presented here is consistent with the observed data, other equivalent models may exist that fit the data as well (Hoyle, 2011, p. 73). The models follow a theoretically reasoned temporal logic, so it is difficult to justify *a priori* why another model should be selected. However, for example, it may be that perceived quality of life influences evaluations of mental and physical health, and it is not possible to eliminate such possibilities in an observational study.

Second, an important source of specification error is the omission of important variables which might influence quality of life. In building structural equation models, it is necessary to balance completeness and parsimony; however, if other variables correlated with those included in the model have been excluded, the estimates of those coefficients may be biased. Such variables known to predict quality of life include cognitive function, depression, or other aspects of the neighbourhood (Llewellyn, Lang, Langa, & Huppert, 2008; Netuveli et al., 2006). A related difficulty is that the mediating variables were not all measured at the same time, and some may have been measured before the moment of retirement. It is possible that the associations between quality of life and variables measured several years before have been weakened as a result of subsequent changes in health and financial circumstances. Although these difficulties might affect the relative importance of the various pathways, they do not affect the main results from this study, that including proximal determinants of quality of life accounts for the association between mid-life social position and quality of life.

Third, the GAZEL study has suffered from attrition, particularly concerning the annual self-completion surveys. The availability of administrative data for participants as well as information provided at baseline means that much is known about the nature of attrition in the cohort, and it is possible to create auxiliary variables that inform on missingness. The use of FIML estimation in this study enables the missing at random assumption (MAR) to be made, rather than the more restrictive missing completely at random (MCAR) assumption required for correct estimation with listwise deletion (Graham et al., 2003). However, it is not possible to verify the MAR assumption and it is possible that data missing not at random have biased the estimates.

Finally, it is conceivable that results from men and women might vary. In order to retain a large sample size for the calculation of path estimates, the genders were analysed together. However, further work in a sample containing a larger number of women could explore the possibility that the pathways linking earlier social position with subsequent quality of life differ between the genders. Additionally, the fact that the female cohort members had careers in gas and electricity distribution, which is unusual, may limit the generalizability of these results to French women as a whole.

## Conclusion

This study has found a finely grained relationship between social position in mid-life and quality of life following retirement. Individuals’ more recent circumstances, in particular their mental and physical health, subjective social status and level of wealth, accounted for this relationship.

These results suggest a minor role for earlier social position in explaining older people's quality of life compared to current circumstances. The measure of quality of life used in this study is closely related to the Third Age. This means that these results suggest, albeit for a population of older people in relatively secure circumstances, that it is individuals’ current situations which overwhelmingly affect their chances of enjoying a period of personal achievement and fulfilment following labour market exit. The policy implication is that earlier disadvantage does not necessarily preclude good quality of life following retirement, and that policies which improve older people's lot, particularly concerning their state of finances and health, might raise their quality of life.

## Appendix

**Table A1.** Covariance matrix and mean values for the variables used in the analysis for the GAZEL cohort study sample (*n* = 11,293).

	1	2	3	4	5	6	7	8	9	10	11	12	13	14	15	Mean
1. CASP-19 (2005)	57.845															43.454
2. Gender	−0.267	0.156														0.193
3. Age-centred	−0.889	−0.171	9.195													−0.003
4. Age-squared	−2.726	0.901	−1.079	101.883												9.195
5. Years since retiring (2005)	−1.490	−0.061	6.550	6.042	10.620											5.264
6. Age at retirement	0.544	−0.120	2.678	−7.344	−4.042	6.942										54.430
7. Mental health (2003)	68.822	−1.350	1.653	−8.529	0.422	1.244	282.942									70.080
8. Physical functioning (2003)	32.753	−0.904	−2.887	−4.787	−3.237	0.299	64.385	164.640								90.144
9. Wealth (2002)	1.551	−0.027	0.191	−0.132	−0.115	0.303	2.128	1.527	1.658							5.647
10. Social status (2004)	2.603	−0.018	0.221	0.019	−0.366	0.572	3.216	2.135	0.591	1.848						4.770
11. Local amenities (2003)	0.434	−0.003	0.018	0.075	0.004	0.015	0.833	0.421	0.024	0.033	0.170					0.781
12. Social support (2004)	0.504	0.003	−0.023	0.056	−0.007	−0.018	0.908	0.209	0.017	0.035	0.007	0.122				0.858
13. Low-mid participation (2005)	−0.164	0.001	0.006	0.059	0.025	−0.020	−0.051	−0.081	0.002	−0.028	−0.004	−0.001	0.239			0.395
14. High participation (2005)	0.513	−0.008	0.011	−0.141	−0.032	0.036	0.513	0.521	0.021	0.065	0.003	0.010	−0.153	0.237		0.387
15. Occupational grade (1989)	3.276	−0.323	1.776	−1.939	−0.372	2.138	6.180	4.746	1.245	2.163	0.003	0.007	−0.059	0.145	10.881	9.037

Note: Variances are displayed on the horizontal diagonal. The sample means presented here were calculated using the FIML estimation procedure and consequently differ from the observed means reported in Table 1.
